# The Marine Viromes of Four Oceanic Regions

**DOI:** 10.1371/journal.pbio.0040368

**Published:** 2006-11-07

**Authors:** Florent E Angly, Ben Felts, Mya Breitbart, Peter Salamon, Robert A Edwards, Craig Carlson, Amy M Chan, Matthew Haynes, Scott Kelley, Hong Liu, Joseph M Mahaffy, Jennifer E Mueller, Jim Nulton, Robert Olson, Rachel Parsons, Steve Rayhawk, Curtis A Suttle, Forest Rohwer

**Affiliations:** 1 Department of Biology, San Diego State University, San Diego, California, United States of America; 2 Computational Science Research Center, San Diego State University, San Diego, California, United States of America; 3 Department of Mathematics, San Diego State University, San Diego, California, United States of America; 4 Center for Microbial Sciences, San Diego State University, San Diego, California, United States of America; 5 Fellowship for Interpretation of Genomes, Burr Ridge, Illinois, United States of America; 6 University of California Santa Barbara, Santa Barbara, California, United States of America; 7 Department of Earth and Ocean Sciences, University of British Columbia, Vancouver, British Columbia, Canada; 8 Argonne National Laboratory, Argonne, Illinois, United States of America; 9 Bermuda Biological Station for Research, St. George's, Bermuda; 10 Department of Microbiology and Immunology, University of British Columbia, Vancouver, British Columbia, Canada; 11 Department of Botany, University of British Columbia, Vancouver, British Columbia, Canada; University of Arizona, United States of America

## Abstract

Viruses are the most common biological entities in the marine environment. There has not been a global survey of these viruses, and consequently, it is not known what types of viruses are in Earth's oceans or how they are distributed. Metagenomic analyses of 184 viral assemblages collected over a decade and representing 68 sites in four major oceanic regions showed that most of the viral sequences were not similar to those in the current databases. There was a distinct “marine-ness” quality to the viral assemblages. Global diversity was very high, presumably several hundred thousand of species, and regional richness varied on a North-South latitudinal gradient. The marine regions had different assemblages of viruses. Cyanophages and a newly discovered clade of single-stranded DNA phages dominated the Sargasso Sea sample, whereas prophage-like sequences were most common in the Arctic. However most viral species were found to be widespread. With a majority of shared species between oceanic regions, most of the differences between viral assemblages seemed to be explained by variation in the occurrence of the most common viral species and not by exclusion of different viral genomes. These results support the idea that viruses are widely dispersed and that local environmental conditions enrich for certain viral types through selective pressure.

## Abstract 2

An extensive metagenomic survey of viral diversity in the marine environment is presented. Many phages are widely distributed, although location-specific selection results in enrichment of some viruses.

## Introduction

Most marine viruses are phages (bacteriophages) that kill the heterotrophic and autotrophic microbes (both Bacteria and presumably Archaea) that dominate the world's oceans [1]. Phages and the other major microbial predator guild, nanoflagellates, control the numbers of marine microbes to a concentration of about ~5 × 10^5^ cells per ml of surface seawater [2,3].

Phages affect microbial evolution by inserting themselves into genomes as prophages. Prophages often account for most of the difference between strains of the same microbial species [4], and they can dramatically change the phenotype of the hosts via lysogenic conversion. For example, many nonpathogens and pathogens only differ by prophages that encode exotoxin genes [5]. Phages also affect microbial evolution by moving genes from host to host. It has been hypothesized that most of the orphan open reading frames (ORFans) in microbial genomes are actually of phage origin [6]. Phages may also affect microbial evolution by killing specific microbes. Various Lotka-Volterra models, called “kill-the-winner,” predict that as one microbial strain becomes dominant, its viral predator kills it and leaves open a niche that can be used by a related strain that is resistant to the phage [7,8]. This model may explain the enormous microdiversity observed in microbial communities [9].

The advent of whole-community genome sequencing (i.e., metagenomics) is rapidly changing the way viral and microbial diversity are assayed. Using this approach, it is possible to rapidly characterize the metabolic diversity and community structure of any microbial ecosystem [10–19]. We studied the marine viral metagenome (virome) of four oceanic regions. The viromes were obtained by pyrosequencing uncultured viral assemblages that were integrated over 4,600 km in distance, 3,000 m in depth, and over a decade in time in order to characterize them and identify patterns of viral distribution and diversity.

## Materials and Methods

### Samples and Sequencing

**Figure 1:**
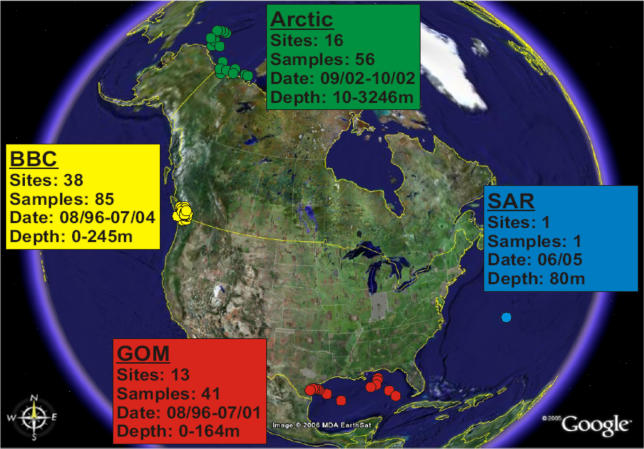
Sampling Sites The circles represent the sampling locations in the Sargasso Sea (SAR), Gulf of Mexico (GOM), British Columbia (BBC), and the Arctic Ocean. The number of samples taken at each location and combined for sequencing, as well as the date and depth range, are shown in the boxes.

Samples were collected from four oceanic regions (Figure 1). Briefly, the viral samples were concentrated on tangential flow filters (30–100-kD cutoff), distributed into 50-ml tubes and stored at 4 °C in the dark. A single sample was collected from the Sargasso Sea (labeled SAR) on 30 June 2005. Chloroform was added to this sample to stop microbial growth. Integrative samples, representing multiple sites and times, were assembled from the Gulf of Mexico (labeled GOM; 13 sites; 42 individual samples), the British Columbia coastal waters (labeled BBC; 38 sites; 85 individual samples), and the Arctic Ocean (labeled Arctic; 16 sites; 56 individual samples). These samples represent the combined viral assemblages of four oceanic regions over approximately one decade (sample details are described in Protocol S1).

Viral particles were purified using a combination of filtration and density-dependent centrifugation ([10]; http://scums.sdsu.edu/isolation.html, accessed 15 September 2006). The cesium chloride gradient was designed to recover virions with densities from 1.35 g ml^−1^ to 1.5 g ml^−1^. Viral DNA was isolated by a formamide/CTAB extraction [20], and the resulting DNA was amplified with Genomiphi and sequenced using pyrophosphate sequencing (454 Life Sciences, Branford, Connecticut, United States) [21] (see Protocol S1 for details on the technology). Each Genomiphi reaction started with 100–150 ng of DNA, above the 10 ng recommended by the manufacturer. A total of 181,044,179 base pairs (bp) of DNA sequence data was generated from the four libraries (SAR, 42 Mbp; GOM, 27 Mbp; BBC, 43 Mbp; and Arctic, 69 Mbp). The difference in library size was due to differences in number of successful reads during the pyrosequencing. The 1,768,297 sequences had an average length of 102 bp. The GOM, BBC, Arctic, and SAR metagenomes are deposited on the SDSU Center for Universal Microbe Sequencing website at (http://scums.sdsu.edu/phage/Oceans, accessed 15 September 2006).

### Bioinformatics

The metagenome sequences from each of the libraries were compared to the SEED nonredundant database and environmental database using BLASTX [22]. The SEED includes the GenBank database supplemented with other complete and draft genome sequences. The environmental database consists of the microbial assemblages from the Iron Mountain acid mine drainage [16], Sargasso Sea [17], whale fall [18], and Minnesota farm soil [18]. All large-scale computational analyses were performed on the Terraport and National Microbial Pathogen Data Resource cluster at Argonne National Laboratory. Individual analyses were performed on a 12-node Orion desktop cluster (Orion, Santa Clara, California, United States).

These comparisons were supplemented with more extensive TBLASTN and with TBLASTX comparisons [22] of either selected portions of the data against the complete nonredundant database or the whole library compared to boutique databases. The same cutoff *E* value was always used for the same database and BLAST search method. In addition, the sequences were compared to the phage and prophage sequences from 510 genomes of the phage genome database (RA Edwards, unpublished data). A FASTA file of these genomes is at http://scums.sdsu.edu/phage/Oceans.

### Taxonomic Composition of the Metagenomes

**Figure 2:**
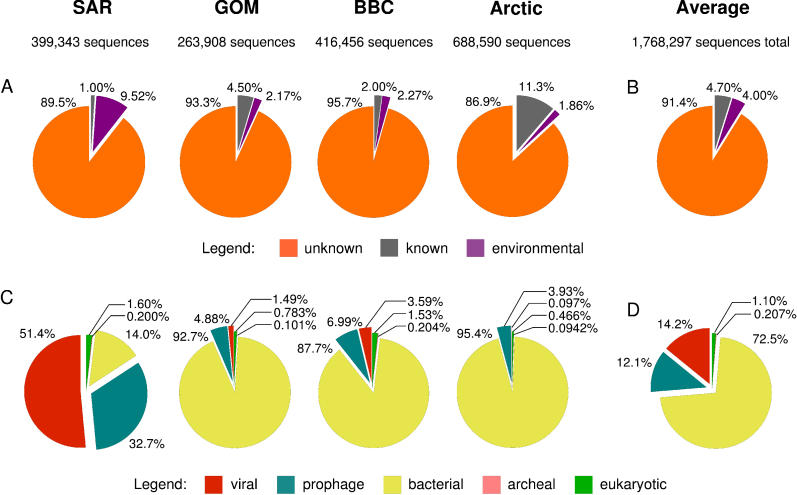
Composition of the Assemblage Genome Sequences as Determined by Similarity to Known DNA and Protein Sequences (A) The percent of “known” sequences compared to the SEED and environmental databases. A sequence was considered “known” if it had a significant similarity (*E* < 10^−5^) to the SEED, else “environmental” if it had a similarity to any environmental database, and else “unknown”. (B) Breakdown of the “known” sequences into viral (both eukaryotic and bacteriophages), prophage, Bacteria, Archaea, or Eukarya.

In an approach similar to previous work [10–12], the best similarity for each metagenomic sequence was automatically parsed and assigned as “known” if there was a significant similarity (*E* ≤ 10^−5^) to a sequence from the nonredundant nucleotide database, else “environmental” for a significant similarity to any environmental database sequence, and else “unknown” (if there was no significant similarity to any database). The number of similarities in each group was then counted (Figure 2A). These numbers were also averaged for the four samples. In a second step, the sequences from the “known” group were classified as viral, bacterial, archaeal, or eukaryotic based on their highest similarity (Figure 2B). To assess the contribution of the prophages (often similar to bacterial sequences), TBLASTX was used to compare the sequences against the complete phage genome sequences. Any significant similarity in the previous four taxonomic groups that was also similar to a prophage sequence was assigned to the prophage group instead. The prophage sequences for these analyses were extracted from complete microbial genomes. A complete list is available at the supporting website (http://scums.sdsu.edu/phage/Oceans). The average of these numbers for the four samples was also calculated.

### Assembly and Verification of Single-Stranded DNA, the chp1-Like Microphage from the Sargasso Sea

The single-stranded DNA (ssDNA) chp1-like microphage was partially assembled from all of the sequences that had significant TBLASTX similarities (*E* ≤ 10^−5^). The assembly parameters were a minimal match percentage of 85% and a 20-bp minimum overlap using Sequencher 4.0 (Gene Codes, Ann Arbor, Michigan, United States). These sequences alone did not result in the assembly of a complete genome due to areas with low similarity to known chp1-like microphage. To complete the assembly, batches of sequences from the Sargasso Sea sample were added to these assemblies until complete coverage was obtained (the consensus sequence is in Protocol S1). The PCR primers SARssDNAF (5′ TGC GGA GAA TAT GGT GAT GA 3′), SARssDNAR1 (5′ CGG TTA TTA CGC CTG TCG TT 3′), and SARssDNAR2 (5′ CCA TGG TAG GGC AGA GGT AA 3′) were designed based on the consensus sequence. A PCR was run against the original Sargasso Sea sample DNA. The reaction mixture (50 μl total volume) contained target DNA, 1 mM of each primer, and 1X FideliTaq master mix (USB, Cleveland, Ohio, United States). The thermocycler conditions were: 5 min at 94 °C; 30 cycles of 1 min at 94 °C, 1 min at 65 °C – 0.5 °C per cycle, 3 min at 72°C; and 10 min 72 °C. Positive PCR products were sequenced for verification of sequence length, and identity was confirmed using TBLASTX.

### Permutation Tail Probability Tests of Phylogenic Similarity between Phage Assemblages

Because the sequences did not originate from a single genetic locus, the evolutionary relationships could not be determined by using standard alignment-based phylogenetic analyses. To determine phylogeny, the sequences were first mapped to the Phage Proteomic Tree based on their best TBLASTX similarity. The version of the Phage Proteomic Tree used here contained 510 complete phage genomes (http://scums.sdsu.edu/phage/Oceans) and was constructed as described previously [23]. Permutation tail probability (PTP) was then used to infer phylogenetic similarity among the phage assemblages. The PTP test uses phylogenetic parsimony to determine whether a given characteristic correlates with phylogeny [24]. Briefly, if a sequence had a best similarity to a phage genome on the Phage Proteomic Tree, it was scored on a tree using Phylogenetic Analysis Using Parsimony software (PAUP) [25]. The number of steps that would be required to produce a tree from one sample to another was then determined. To assign significance, this value was compared to a distribution produced by randomizing the input tree 10,000 times.

### Genetic Isolation by Distance of the Phage Assemblages

Isolation by Distance Web Service (IBDWS) ([26]; http://biome.sdsu.edu/ibdws) was used to test for a correlation between the geographic distance between two samples and the genetic divergence between viral assemblages. This online software uses Mantel tests to determine whether marine phages in closer physical proximity have greater genetic similarity (as measured by Φ_ST_) than those separated by large geographic distances. For these tests, the current datasets were combined with data from the California coast [10]. The Arlequin program [27] was used to calculate Φ_ST_. The Φ_ST_ statistic compares the phylogenetic diversity within each assemblage to the total phylogenetic diversity of the combined assemblages using the equation:

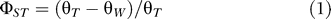
where θ_T_ is the total phylogenetic diversity of two assemblages and θ_W_ is the phylogenetic diversity within each assemblage or population. A Φ_ST_ value close to zero means there is complete overlap in the phylogenetic diversity, whereas values greater than zero indicate increasing levels of phylogenetic differentiation up to a value of 1, indicating complete differentiation.

### Assembly and Mathematical Modeling of Viral Assemblage Diversity

To estimate viral diversity, sets of 10,000 random sequences from each oceanic region were assembled using TIGR Assembler [28] with a minimum overlap length of 35 bp, a minimal match percentage of 98% and no alignment error in 32 bp to identify overlapping sequences (contigs) [10]. The Perl script used to automate this task is available at http://scums.sdsu.edu/phage/Oceans. Average contig spectra were calculated (Figure S3) over ten repetitions, and the maximum likelihood assemblage structure of the marine viral assemblages was determined using mathematical rank-abundance models in PHAge Communities from Contig Spectra (PHACCS) ([29]; http://biome.sdsu.edu/phaccs). Random subsamples of the metagenomes were used instead of the totality of the whole metagenomes, because PHACCS analyses are more robust at low coverage [10,11,29]. The diversity estimates for the best-fitting assemblage model were used for each oceanic region. Detailed graphical explanations of these procedures are given in Protocol S1.

To analyze the degree of similarity between the viral assemblages, the amount of overlap between the assemblages was determined by assembling a mixed sample of 10,000 fragments obtained by pooling 2,500 fragments from each region. The fact that fragments from one region assembled with fragments from another region indicates overlap between the metagenomes of the two regions, and the extent of this overlap quantifies the similarity. The contig spectrum obtained from the mixed sample was modified in two respects to give what is called the cross-contig spectrum (Figure S4). First, any contig that contained fragments exclusively from a single region was removed (i.e., only contigs that included fragments from more than one region were counted). Thus for the contigs of size *q* > 1, 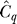 , the number of *q*-contigs from the pooled sample that included fragments from more than one region, was calculated. Second, the number of 1-contigs from each region that assembled with any fragments from other regions was used as the number of 1-cross-contigs, 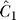 . The resulting cross-contig spectrum 
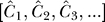
 was then compared to the mean cross-contig spectrum from simulated mixtures of the four assemblages. To simulate such mixtures requires a model of which genomes with a certain rank and abundance in one assemblage correspond to which genomes in another.

There are many ways to envision morphing one assemblage of genotypes (species defined on the genomic level by assembly of sequences) into another. For these analyses, two morphing modes were considered (Figure S5): (i) varying the proportion of genotypes that were shared between assemblages and (ii) varying the proportion of the genotypes whose abundance ranks were shuffled (i.e., subjected to a random permutation). Using these two degrees of freedom, *s* (percent shared) and *p* (percent permuted), Monte Carlo analyses were performed to estimate the degree of morphing as measured by these two parameters to find maximum-likelihood values for *s* and *p* based on the closeness of the match to the cross-contig spectrum found for the pooled sample.

The Monte Carlo simulations were all performed using the best-fit models for each region. The cross-contig spectrum based on the mixed sample was used to perform these simulations (Figure S6). Each simulation included 861 pairs of *s* and *p* values spanning a 21 × 41 grid between 0% and 100% for each parameter. Each simulation randomly permuted the abundance rank of *p* of the most abundant genotypes, randomly assigned *s* of the genotypes to be shared, and determined the resulting predicted cross-contig spectrum. This was repeated 100 times for each combination of *s* and *p* values. The entire simulation, including the selection of the 2,500 fragments from each region, was repeated eight times resulting in 800 predicted cross-contig spectra for each combination of parameter values. The mean 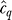 and variance 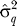 of these 800 values were then used to construct a quasi-likelihood 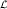
(*s,p*)

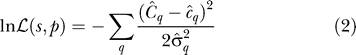
of matching the observed cross-contig spectrum, thereby generating a contour map of L as in [11]. This log likelihood would be expected if each cross-contig value were normally distributed. The contour map of the quasi-likelihood landscape was produced from this grid of 861 quasi-likelihood values. As a control, the whole procedure was repeated for all regions with nonoverlapping subsets of sequences all taken from the same geographical region (rather than from four different regions).

## Results/Discussion

“Community” is commonly defined several ways, including “the species that occur together in space and time” [30] and “an association of interacting populations” [31]. Assemblage is probably the most proper term to describe viral groups, and most instances of “community” in the literature, both by ourselves and others, is not correct. See [32] for a disambiguation of some important ecological terms.

### General Characteristics of the Marine Viral Metagenomes

On average, >91% of the sequences were not significantly similar to those in the extant databases (Figure 2A). A partial explanation for the high percentage of unknowns is almost certainly due to the shorter sequences (~100 bp on average) that are generated by pyrosequencing at 454 Life Sciences. Previous viral metagenomic studies that used Sanger sequencing (~650 bp fragments) found that >60% of the sequences were unknowns [33]. The Arctic Ocean sample had the highest percentage of known similarities (11%) to the SEED database, mostly because of the large number of prophage-like sequences (Table 1). Comparison of the marine viral sequences to the environmental database did not yield a significant number of new similarities compared to the SEED database (~2% to the environmental database), with the notable exception of the Sargasso Sea sample, where >9% of the similarities were to the environmental database, presumably because the major sources of sequences for the environmental database were the Sargasso Sea microbial metagenomes, originally collected in 2003 [17]. The overlap between the viral metagenome and the microbial metagenomes raises several important points. First, a significant number of viral sequences are retained on the larger-pore filters, either as free viruses, proviruses, or in cells undergoing a burst. The latter explanation was hypothesized by Delong et al. [19], who observed a large number of viral similarities at one depth at the Hawaii Oceanic Time-series (HOT) station. Second, the microbial assemblages in the Sargasso Sea appear to be relatively stable over prolonged periods (~2 y). Finally, the small amount of sampling and sequencing represented by these two studies (~10^12^ bp) is already constricting the unknown sequence space of the Sargasso Sea. With the continual decline in Sanger sequencing costs and introduction of large-scale pyrosequencing, metagenomic approaches should be able to characterize global sequence diversity in a relatively short period of time.

**Table 1:**
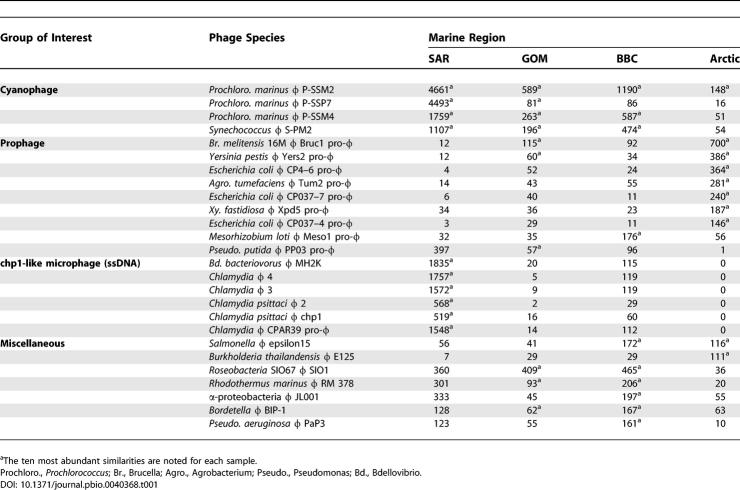
Number of Similarities to Phage Genomes and Groups of Interest in the Four Metagenomes

Among the fraction of sequences with similarity to the SEED database, most of the “knowns” were similarities to bacterial sequences in the Arctic, British Columbia, and Gulf of Mexico samples (Figure 2B). This can be accounted for by the following: (i) the larger number of microbial rather than viral genomes in the database, (ii) unidentified prophages within microbial genomes, (iii) the large amount of horizontal gene transfer between phages and their hosts, (iv) phages carrying full genes from their host, as observed in sequenced phage genomes [34,35], and (v) the overall larger size of bacterial genes relative to viral genes, statistically increasing the probability of sequencing and hitting them.

**Figure 3:**
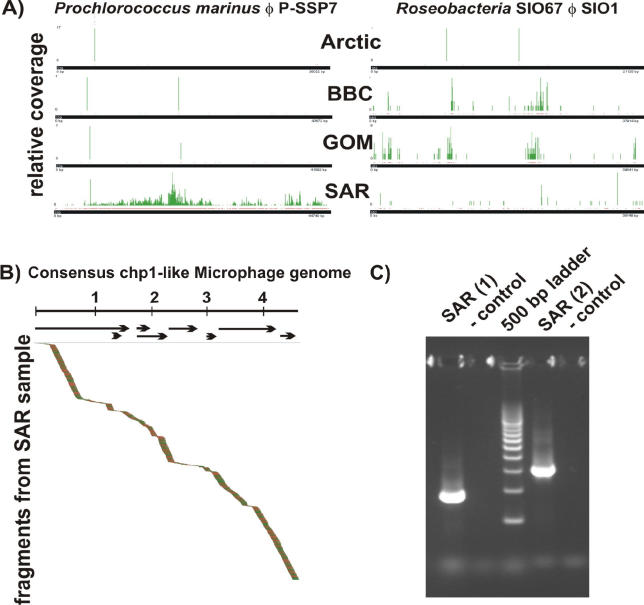
Distribution of Similarities and Assembly Controls (A) Distribution of similarities between the four metagenome samples to the P. marinus φ P-SSP7 and *Roseobacteria* SIO67 φ SIO1 genomes (as determined by BLASTN analysis). The green bars represent the average number of sequences averaged over 100 bp windows. (B) Comparison of fragments from the Sargasso Sea metagenome against the consensus ssDNA chp1-like microphage genome. The consensus from this assembly is in the Protocol S1. (C) PCR verification of chp1-like microphages in original SAR sample. PCR primers were designed based on a consensus sequence from the assembly shown in (B). SAR1 is a ~900-bp fragment and SAR2 is a ~1,500-bp fragment.

The sample from the Sargasso Sea was exceptional in that the majority of “known” sequences were most similar to three *Prochlorococcus* phage genomes (Table 1) originally isolated from the same area of the ocean [34]. This finding suggests that just a few phage genomes from novel environments will greatly increase our understanding of viral diversity in these environments. The distribution of BLASTN similarities along the Prochlorococcus marinus φ P-SSP7 genome [34] is shown in Figure 3A. There is almost complete coverage of the genome within the Sargasso Sea sample. In contrast, the similarly sized *Roseobacteria* SIO67 φ SIO1 genome [36], which was isolated from near-shore waters in California, is only sparsely covered in the Sargasso Sea sample, but has higher coverage in the Gulf of Mexico and British Columbia samples. This supports the idea that certain phage groups are more prevalent in certain biogeographic regions. This general pattern was reinforced by the observation of a number of phage genomes and groups prevalent in different oceanic regions (Table 1).

The five most abundant putative viral-encoded enzymes (Table 2) appear to be involved in scavenging host nucleotides (e.g., riboreductases) and supporting host metabolism through the infection cycle (e.g., carboxylyases and transferases). The viral fraction also contained *psbA* genes, which encode the D1 protein of photosystem II in the cyanobacteria. The majority of sequenced cyanophages carry this gene, and evidence is mounting that the cyanophages need the D1 protein for successful infection and replication [34,37,38]. The occurrence of *psbA* was lowest in the Arctic sample, probably reflecting a decrease in the host and cyanophage numbers in the colder environments.

**Table 2:**
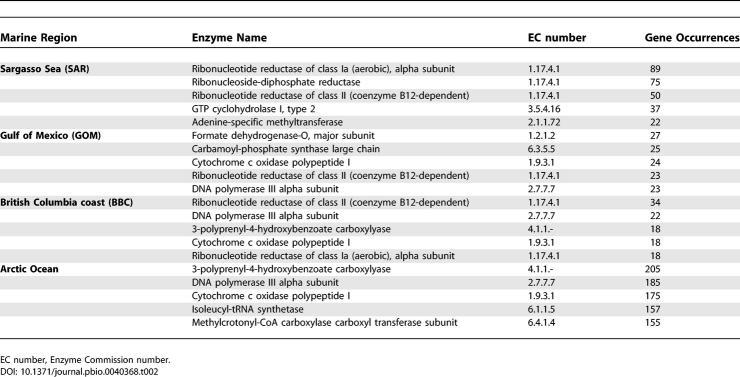
The Most Abundant Enzyme-Coding Genes in the Four Oceanic Viral Metagenomes

### Discovery of an Abundant Marine ssDNA Phage Group

**Figure 4:**
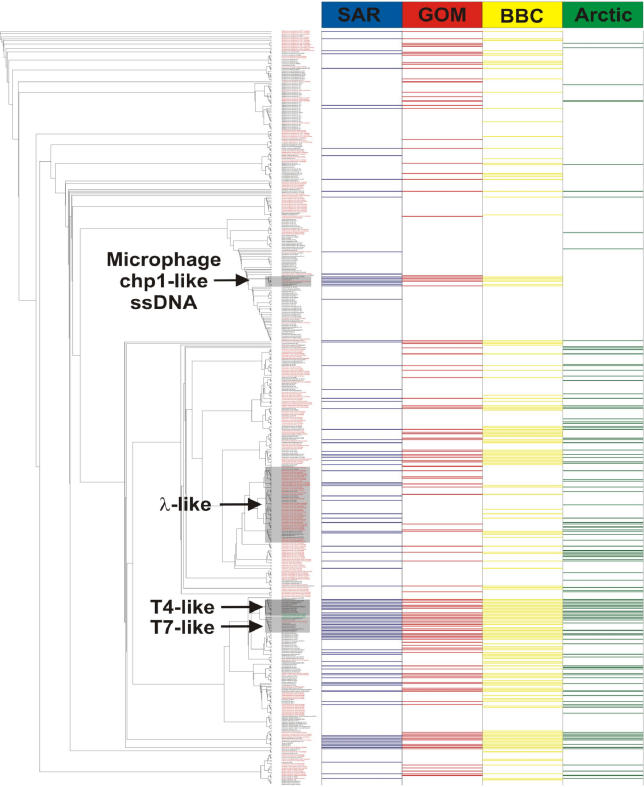
Types of Phages in the Four Metagenomes A new version of the Phage Proteomic Tree (left panel) was constructed from 510 complete phage and prophage genomes using the previously described method [23]. The metagenomic sequences were compared to the phage on the Phage Proteomic Tree using TBLASTX, and the colored bars on the right represent significant similarities (*E*-value < 0.0001). Names of prophages are in red and the *Prochlorococcus* phage genomes are in green. An electronic version of the tree and a FASTA list of phage and prophage genomes used to make the tree are available at the SDSU Center for Universal Microbe Sequencing website (http://scums.sdsu.edu/phage/Oceans).

The Sargasso Sea sample had a large number of sequences (6% of the total; Table 1) with significant similarities to chp1-like Chlamydiamicrovirus (Microviridae family). These viruses are small ssDNA phages. Assemblies from these sequences resulted in the near-complete genomes of several marine Microviridae phages from the Sargasso Sea sequences (Figure 3B). To our knowledge, this is the first report describing the presence of this phage group in the marine environment, which was previously overlooked because the amplification and cloning methods excluded ssDNA viruses. The only other report of ssDNA viruses in the marine environment was a Circovirus that infected diatoms [39]. However, the marine sequences in this study did not show any similarity to that virus. Sequences with significant similarity to the chp1-like phages were observed less frequently in the British Columbia (~10-fold less common than in SAR) and Gulf of Mexico samples (~100-fold less common than in SAR). No sequences from this group were found in the Arctic sample (Table 1 and Figure 4). Primers were designed against these genomes and appropriately sized DNA fragments were amplified from the Sargasso Sea sample (Figure 3C). No amplicons were detected in the Gulf of Mexico or British Columbia samples, suggesting that they were present at numbers below the level of detection in this PCR or had a divergent sequence. A geographical constraint that limits the distribution of these viruses would be most consistent with these results. However concerns about sample amplification and storage bias make it impossible to accurately access the relative abundances of these viruses at this point.

### Every Phage Everywhere?

The distribution of similarities to the chp1-like Microphage, P. marinus φ P-SSP7, *Roseobacteria* SIO67 φ SIO1, and others in the viral-fraction suggests that viruses have restricted geographical distributions similar to those observed in micro- and macro-organisms [40,41]. This is in contrast to studies that have shown that identical phage genes are distributed throughout the biosphere and that phages from soils and sediments can replicate in marine microbial populations [3,42,43]. To determine whether all marine phages are spread everywhere or if there is a strong regionalization, three different approaches were used.

A new version of the Phage Proteomic Tree was constructed, and similarities from the samples were mapped onto this tree (Figure 4). Eighty-four phage species were specific to one marine region, whereas 45 were common to all four. From the remaining phage species, 102 were found in several oceanic regions. The phylogenetic parsimony of phages from each sample was compared to the Phage Proteomic Tree using the PTP tests, because viruses do not have a single genetic locus conserved across all genomes. The PTP test showed that the distribution of phages in the marine samples is not random. First, marine phages are phylogenetically distinct from the available genomes, suggesting a “marine-ness” to the group as a whole (*p* < 0.0001; 10,000 randomizations). Second, there was a significant difference between phages from the different oceanic regions (*p* < 0.0001; 10,000 randomizations), supporting a geographical specificity for viruses despite the wide prevalence of some phage species.

**Figure 5:**
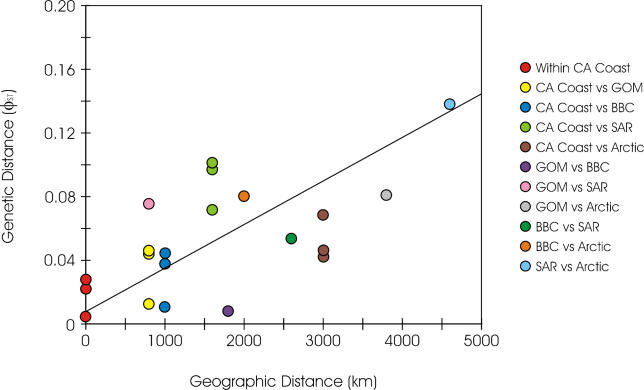
Relationship between Geographic and Genetic Distances of Marine Viral Assemblages In addition to the four metagenomes sequenced for this study, the previous viral metagenomes from the San Diego area (California coast) were also included in this analysis [10]. There was a significant correlation of 3.28 × 10^−5^ Φ_ST_ / km (Mantel test, *Z* = _−_78.9, *p* < 0.017, *r* = 0.585).

An Isolation By Distance (IBD) approach demonstrated that there was a significant positive correlation between geographic distance (km) and genetic distance (as measured by Φ_ST_) (Mantel test; *Z* = −78.9; *r* = 0.585; *p* < 0.017) (Figure 5), indicating that the further two sites are from each other, the more differences there are between the viral assemblages. The magnitude of the slope was very small with only 3.28 × 10^−5^ Φ_ST_/km.

Considering that any two locations on Earth can be separated by a maximum of 20,000 km (half the circumference of the globe), by extrapolation, any two viral assemblages could have a phylogenetic diversity of at most 0.656 Φ_ST_. Although these data suggest a limit to the distribution of viruses among marine environments (e.g., due to limited viral movement or geographical selective pressure) (Φ_ST_ >> 0), it also indicates that no two marine viral assemblages could be totally different (Φ_ST_ << 1). Rather, they would exhibit a relatively large phylogenetic overlap.

Together the PTP and IBD test support that the marine virome is composed of specific viral groups. These viral assemblages undergo a regionalization, although a large fraction is vastly widespread. It is possible that some viruses are distributed ubiquitously, but their relative contribution to overall assemblage structure differs between oceanic regions. If this were true, then cross-contigs—i.e., contigs made of sequences from different metagenomes—would reflect this composition.

In the computer model of cross-contig analysis, all four viral assemblages were considered at the same time. Assemblies were performed and cross-contigs were identified. A Monte Carlo simulation was used to explain the average cross-contig spectrum. A full description of the assemblies and Monte-Carlo simulations are in the Protocol S1.

**Figure 6:**
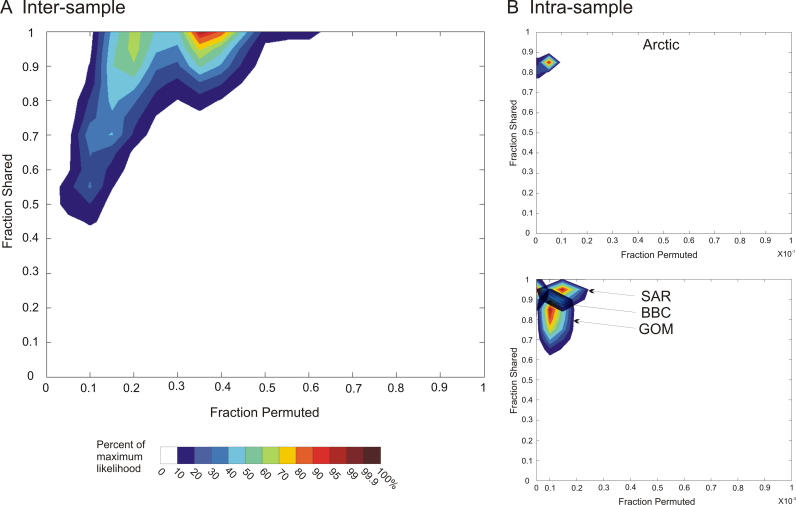
Monte Carlo Simulation of Cross-Contigs between Metagenomic Samples (A) For the intersample analysis, the maximum likelihood occurred at 35% fraction permuted and 100% fraction shared. (B) The maximum likelihood was between 0% and 0.5% fraction permuted and 85% and 95 % fraction shared for the intrasample controls.

A number of genotypes (varied between 0% and 100%) were arbitrarily and randomly defined as shared between samples; at the same time, the occurrence of individuals in the viral assemblage was also varied (Figure 6). As an illustration, imagine two assemblages sharing 100 viruses, but with the relative rank on a rank-abundance curve being shuffled for the top viruses in the assemblage (see Protocol S1). The best explanation of the observed cross-contigs is shown in Figure 6 and estimates that 35% of the most abundant genomes in any sample would have to be permuted in their relative abundance rank and that 100% of the viruses would have to be shared between samples. The intrasample controls showed that 85%–95% of the most abundant genomes were shared and 0%–0.5% were permuted (although 100% and 0% were expected, respectively). This discrepancy is probably due to limitations in the methodology used.

This cross-contig analysis suggests that any two viral assemblages could have a vast majority of species in common and the order of the ranks in the rank-abundance curve could be determined by shuffling about a third of the most abundant species. These results confirm that geographical and changing environmental conditions allow different viral genotypes to become more or less prevalent within different assemblages while sharing essentially the same types of viruses. The less abundant viruses are not lost altogether, merely reduced in occurrence.

### Local Versus Global Diversity

Using the PHACCS analysis system [29], the genotype richness, diversity, and evenness of the different metagenomes were estimated (Table 3). The British Columbia viral metagenome was the most genotype-rich (129,000 predicted genotypes) and diverse (H′ of 10.8 nats), whereas the Arctic metagenome was the least genotype-rich (532 predicted genotypes) and diverse (H′ of 6.05 nats).

**Table 3:**
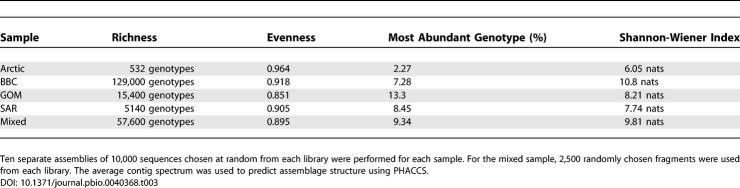
Viral Assemblage Structure Predicted from Assembly of Metagenomic Sequences

Being located on the west coast of the North American continent, the coast of British Columbia is in an upwelling area. It is also enclosed and fed by many rivers. These conditions might importantly increase the diversity of microbial communities and thus provide an explanation for the very high viral assemblage diversity estimated in this oceanic region. Omitting the BBC, the viral diversity of the other regions (the Gulf of Mexico, Sargasso Sea, and Arctic Ocean) correlate with the well-established North-South latitudinal diversity gradient [44], with a larger diversity at lower latitudes. Planktonic diversity patterns of near-shore versus off-shore (more diverse plankton assemblages off-shore) [45] were not observed here; the large spatial scale of the sampling probably masked this effect if present.

Assemblies of the mixed sample were used to predict global viral diversity using PHACCS. A total of 57,600 different viral genotypes in all four regions (H′ of 9.8 nats) was estimated. This number is smaller than the number of genotypes predicted in the BBC sample, which may indicate an undersampling for the mixed metagenome or be due to some of the assumptions of the model. Taken together, these data indicate that the global marine viral richness could be as high as a few hundred thousand species, with a regional richness sometimes almost as high, likely because of migration processes.

### Integrative Versus Single Samples

It was expected that the integrated samples would be more even because it is assumed the viruses that were most abundant at one spatial-temporal time point would be rarer at another (“kill-the-winner” hypothesis). As summarized in Table 3, the evenness of the single time point sample (SAR 0.905) fell in between that of the three integrated samples (Arctic 0.964; BBC 0.918; GOM 0.851). Similarly, the predicted richness (5140 genotypes) and diversity (H′ 7.74 nats) at the single point represented by the Sargasso Sea sample fell in between that of the integrated samples (richness 532–129,000; H′ 6.05–10.8 nats). Because of factors with a supposedly greater impact, like latitude, it is not clear that integrating individual samples gave a greater depth of coverage.

Without a doubt, many interesting trends based on depth and a wide variety of other spatial, biological, and temporal parameters were missed by the integrative sampling used here. However, this sampling does provide a useful overview of the marine virome on a global and regional scale. Currently, there are no real criteria as to what constitutes a useful size or time scale for sampling natural viral assemblages, so there is no particular advantage or disadvantage to keeping samples separate or analyzing them as a metadataset. Rather the sampling scheme should be driven by the question being addressed. Viral assemblages are interesting in their own right, not just in context of their host communities. However, future studies should also start cross-correlating the viruses with their hosts. Of particular interest will be determining if the “islands” and ORFans observed in microbial genomes are represented in the virome [6,46].

### Potential Sampling and Processing Biases

Sampling bias in the current datasets was primarily due to loss of large viruses during filtering. Currently, there is no experimental method to avoid this problem. The cesium chloride gradients used here recover all known phage groups, and essentially all the viral-like particles in the starting samples migrate to the proper density in these preparations (as observed by epifluorescence microscopy; unpublished data). Unfortunately, the cloning methods used here will not recover RNA viruses. Suttle et al. [47,48] have shown that RNA viruses are present in the marine environment. Whereas most electron microscopy [49,50] and nucleic acid–based studies [51] have not found RNA viruses in large numbers, RNA viruses are still believed to be important components of the marine virome that need additional study.

Another potential source of bias is the different times that the samples were stored before processing. Phage particles are very stable and often stored for decades at 4 °C. This is a commonly known lab phenomenon and is supported by the observation that the oldest viral concentrates (~12 y old) in this study had very high concentration of viruses (>10^9^ viral-like particles per ml). Different phages, however, may have different decay rates under these conditions. This does not seem to be especially problematic, because there is no correlation between the types of viruses observed and the storage time. For example, the Arctic and SAR samples are the most recently harvested samples, yet they have the biggest differences in terms of types of phages (Table 1). Nonetheless, there may be effects of storage on the composition of the viral assemblages. For this reason, analyses based on absolute abundances of one specific virus to another were avoided in this study. Instead, the presence of a sequence in the metagenome was simply assumed to mean that the virus was in the original sample (i.e., an occurrence).

Whole-genome amplification techniques introduce biases in the relative concentrations of different genomes. Tests of Genomiphi by the manufacturer and others [52,53] have not reported a significant bias in the amplification of circular double-stranded DNA (dsDNA), with the exception of very small dsDNA targets (<1 kb), which are much smaller than the vast majority of marine viruses, and of ssDNA, which will probably be a preferred target for the DNA polymerase. Although not bias-free, Genomiphi is the most accurate amplification method available [54]. Interesting trends associated with viral assemblage structure may have been missed because of our choice of using presence/absence data for the analyses presented here, but by being conservative there should not be any effects of storage, amplification, and sampling biases on our interpretations.

## Conclusion

The metagenomic analysis of viral assemblages from the Arctic Ocean, the coast of British Columbia, the Gulf of Mexico, and the Sargasso Sea presented here has changed our perception on the composition of viral assemblages in the sea. First, there is clear evidence that the composition of viral assemblages varies in different geographic regions probably reflecting selective pressure. Previously overlooked viral groups, such as ssDNA viruses and prophages, can be major constituents of marine viral assemblages (Sargasso Sea and Arctic Ocean, respectively). Second, global viral diversity is high (possibly a few hundred thousand viral species), but regional diversity can be almost as high due to viral migration. This migration provides opportunities for global exchange of DNA among viral genomes, as predicted by the mosaic model [55]. Viral diversity also varied according to latitude, with a higher richness at low latitudes. Finally, it seems that although some viral species are endemic and others are ubiquitous, the vast majority are widespread and shared between several oceanic regions. Invasion and replacement by new phages does not appear to be an important structuring factor for these viral assemblages. What sets different assemblages apart is likely the change in abundance of its most abundant members, supporting to some extent the old tenet “everything is everywhere, but, the environment selects” [56] for marine viruses.

## Supporting Information

Figure S1 Frequency of Homopolymeric Tracts in the Four Marine Viromes, the Complete Phage Genomes, and Twenty, Randomly Chosen Microbial Genomes The tracts from 3 nucleotides (nt) to 15 nt were counted and normalized to the number of bases in each sequence. One 3-nt tract is found approximately every 30 bp, whereas one 15-nt tract is found approximately every 10 million bp. The 510 complete phage genomes totaled 18,909,173 bp in length, and the microbial genomes totaled 22,110,123 bp in length. The lengths of the pyrosequenced libraries are given in the text.
(1.3 MB TIF)

Figure S2 Relative Abundance of Phages in the Four Metagenomes Because of way the samples were stored and the long storage time, the distribution shown may not accurately reflect the reality.
(104 KB TIF)

Figure S3 Determining a Normal Contig Spectrum (135 KB TIF)

Figure S4 Getting a Cross-Contig Spectrum. (3.1 MB TIF)

Figure S5 The Possible Scenarios Considered in the Monte Carlo Simulation to Explain the Observed Cross-Contigs (143 KB TIF)

Figure S6 Analyzing a Cross-Contig Spectrum (589 KB TIF)

Protocol S1 Details on Materials and Methods. (39 KB PDF)

### Accession Numbers

The Genome Projects Database (http://www.ncbi.nlm.nih.gov/Genomes) accession numbers for the sequences are 17765 (GOM), 17767 (BBC), 17769 (Arctic), and 17771 (SAR); the Genome Catalogue (http://gensc.sf.net) accession numbers are 000002_GCAT (GOM), 000003_GCAT (BBC), 000004_GCAT (Arctic), and 000005_GCAT (SAR); and the GOLD database (http://www.genomesonline.org) GOLDstamps are GM00060 (GOM), GM00061 (BBC), GM00062 (Arctic), and GM00063 (SAR).

## Abbreviations

bp: base pair
PTP: permutation tail probability
ssDNA: single-stranded DNA
