# Supplementary material for: The Marine Viromes of Four Oceanic Regions
Source: PLoS Biol. 2006 Nov 7;4(11):e368. doi: 10.1371/journal.pbio.0040368 (PMC1634881; doi:10.1371/journal.pbio.0040368)
Supplement: Supplementary file 1 [file pbio.0040368.sd001.pdf]

Supplementary Material for "Marine Viromes of Four Oceanic Regions"
by Angly et al.

Sample collection and preparation

Field collection from the Sargasso Sea (SAR): Samples were collected on June 30, 2005 from Hydrostation S (32° 10' N, 64° 30' W) located in the northwestern Sargasso Sea approximately 26 km southeast of the island of Bermuda, essentially the same spot the Surge Samples were collected. Water from 80 m was retrieved via the *R.V. Weatherbird II's* CTD rosette equipped with 12 liter Niskin bottles. This depth was targeted because summer subsurface maximum in viral like particles (VLP) is historically located between 80 and 100 m. Upon recovery of CTD, seawater was transferred to a pre-cleaned 150 L polypropylene carboy via acid washed silicone tubing. Viruses were concentrated using a Pelicon II tangential flow filtration system (Millipore Corporation; Bedford, MA) equipped with a 30 kd Biomax cassette filter (0.5 m^2; modified polyethersulfone). Prior to collection the filter was cleaned with 0.1 N H_3P0_4 recirculated for 45 minutes followed by 0.1N NaOH recirculated for 45 minutes. One-hundred and fifty liters of seawater was recirculated over the 30 kD filter until the retentate volume was ~150 ml. This concentration step took ~3 hours to complete. The final concentrate was distributed into four 45 ml aliquots in sterile Falcon tubes. Five ml of chloroform was added to each tube, stored and shipped at 4° C until arrival at SDSU. The final viral concentration was approximately 1.4 X 10^9 VLP ml^{-1}.

Field collection from the Gulf of Mexico (GOM), the Bay of British Colombia (BBC), and the Arctic: Samples were collected from the Gulf of Mexico, the Arctic Ocean, the Strait of Georgia, British Columbia and adjacent inlets, and Barclay Sound on the west coast of Vancouver Island. Further details on the samples and locations are listed in Supplemental Table S1. Seawater samples (10 to 200 liters) were prefiltered through 142 mm-diameter glass fiber filters (1.2 µm nominal pore-size: type GC50, Advantec MFS, Dublin, CA, or 0.7 µm nominal pore-size: type GF/F, Whatman, Clifton, NJ) followed by either 0.45-µm-pore-size (type GVWP, Millipore, Bedford, MA) or 0.2 µm-pore-size (Gelman, East Hills, NY) membrane filters. Virus-sized particles in the filtrate were concentrated ca. 50- to 700-fold by ultrafiltration using a 10 or 30 kDa-cutoff Amicon/Millipore (S1Y10/S1Y30/S10Y30) spiral cartridge and then stored at 4°C in the

dark. One milliliter of each virus concentrate (VC) was combined into one of the following mixes based on its geographical origin. The Gulf of Mexico mix consisted of 41 different virus communities (3.65×10^{10} vlps per ml), the Arctic mix consisted of 56 different virus communities (1.11×10^{10} vlps per ml), while the British Columbia mix consisted of 85 different communities (3.4×10^{10} vlps per ml). Virus abundances in each mixture were counted using SYBR Green I (Invitrogen, Carlsbad, CA) and epifluorescence microscopy.

Table S1. Temporal and spatial sampling of 4 marine provinces.

Location	Number of Stations	Number of Samples	Sampling Dates	Salinity (psu)	Temp (°C)	Depth (m)
SAR						
Hydrostation S	1	1	6/05			80
GOM						
Western GOM	2	6	6/95 and 7/96	36.2 to 36.4	20.2 to 30.5	surface to 164
Texas Coast	5	13	6/94 to 7/96	26.1 to 40.6	14.7 to 30.5	surface to 5
Northeast GOM	6	14	7/01	31.5 to 36.6	18.7 to 30.0	1 to 120
Eastern GOM	2	8	7/01	33.5 to 36.6	21.6 to 30.0	3 to 90
Arctic						
Chukchi Sea	7	14	9/02	26.8 to 35.0	-1.4 to 5.4	10 to 3246
Canadian Arctic [Beaufort Sea, MacKenzie Shelf, Amundsen Gulf]	23	42	9/02 to 10/02	20.3 to 34.9	-1.5 to 1.5	2 to 968
BBC						
Strait of Georgia and adjacent inlets, Barclay Sound	38	85	8/96 to 7/04	14.0 to 31.3	7.0 to 22.6	surface to 245

Preparation of Marine Phage DNA samples for pyrosequencing at SDSU: Viral concentrates were filtered with 0.22 µm Sterivex cartridge filters to remove any microbial contaminants. Viral particles were treated with DNase, RNase, and purified by cesium chloride (CsCl) gradient centrifugation. Approximately 8.5 ml of viral concentrate, with CsCl added to create a density of 1.15 g ml^{-1}, was layered onto a step gradient comprised

of CsCl solutions at 1.7 g ml^{-1}, 1.5 g ml^{-1}, and 1.25 g ml^{-1}. CsCl solutions were made up in filtered and autoclaved seawater, obtained separately from the samples. The gradients were centrifuged at 22,000 rpm in an SW41 swinging bucket rotor at 4° C for 2 hours. After centrifugation, the 1.5 ml corresponding to the 1.5 g ml^{-1} gradient step plus the interfaces above and below, were withdrawn from the tubes with a syringe and a 18 gauge hypodermic needle.

DNA was extracted by addition of buffers to yield final concentrations of 0.2 M Tris, pH 8, and 5mM EDTA, followed by addition of 1 volume of deionized formamide. These samples were then incubated at room temperature for 30 min, after which 2 volumes of 100% ethanol were added to precipitate the DNA. The DNA was pelleted by centrifugation at 12,000 rpm in a fixed-angle rotor at 4° C for 20 min. The pellets were washed with 70% ethanol, and then resuspended in 567µl TE buffer (10 mM Tris, pH 8.0, 1mM EDTA) and then 30 µl of 10% SDS (sodium dodecyl sulfate) and 3 µl of proteinase K (20 mg ml^{-1}) were added, and the samples were incubated at 37° C for 1 hour. Subsequently 100 µl of 5 M NaCl and 80 µl of CTAB NaCl solution (0.7 M NaCl, 10% w/v cetyl trimethyl ammonium bromide) were added, followed by incubation for 10 min. at 65° C. The samples were then extracted with 1 volume of chloroform, then with 1 volume of phenol:chloroform:isoamyl alcohol (1:1:24), and finally with 1 volume of chloroform, after which 0.7 volumes of isopropyl alcohol (2-propanol) were added to precipitate the DNA. After storage overnight at -20° C, sample tubes were centrifuged at 12,000 rpm in a microcentrifuge at 4° C. The pellets were washed with 70% ethanol, dried and resuspended in 50 µl H_2O.

The DNA was amplified with Genomiphi kits (Amersham; Ø-29 DNA polymerase) for 18 hours in a thermal cycler, using multiple 20 µl reactions containing 50-100 ng of the isolated DNA as template. After amplification, the resulting DNA was purified with silica columns (Qiagen) to remove the enzyme, dNTPs, and primers, then ethanol precipitated and resuspended in H_2O to yield a DNA concentration of ~0.3 mg ml^{-1}. DNA samples (~10 µg each) were sequenced using pyrophospate sequencing technology (454 Life Sciences, Inc, Branford, CT).

454 pyrosequencing

Potential errors associated with 454 pyrosequencing: There are two main concerns associated with pyrosequencing: 1) random errors, where an incorrect base is substituted for a correct base, and 2) systematic errors, due to homopolymeric runs (i.e., runs of the same base). Since none of the marine virome sequences were known, the random error rate can not be directly determined from the data. It is assumed that the error rate is approximately the same as other investigators are reporting from very deep coverage of known sequences (e.g. primers included in the sequence). In these cases the error rate seems to be much less than 1 incorrect base in 1,000 reads (Edwards, personal communication).

454 Life Sciences, Inc, assert that their sequencing technology is accurate up to at least 8 homopolymeric nucleotides. To test this assertion, and to estimate the effect of these errors on the sequence analysis performed here, the frequency of homopolymeric runs from 3 nt to 15 nt were calculated for each of the four marine viromes, a database of 510 complete phage genomes, and 20 complete microbial genomes (Figure S1).

In general the marine virome contained very similar numbers of homopolymeric tracts as the microbial genomes. For unknown reasons there appear to be less 9-mers through 13-mers in the completed phage genomes than in either the microbial genomes or the viral libraries sequenced here. No 14 nt homopolymeric tracts were found in any of the 510 complete phage genomes. Presumably the higher packing density of genes in phage genomes, and the decreased information contained in long homopolymeric tracts is selected against in these genomes. In contrast to the rumored problems with homopolymeric tracts, this analysis seems to demonstrate that there are about as many tracts in 454 pyrosequenced databases as in complete bacterial genomes, and in fact the Sargasso sample sequenced here appears to contain a few more of these tracts than other databases.

In total, 15,543 sequences containing homopolymeric tracts between 9 and 15 nt were found in the four libraries (Table S2). Therefore, less than 1% of the sequences contain a homopolymeric tract that would be susceptible to the compression error of concern with pyrosequencing. We therefore conclude that the errors associated with

compression of consecutive nucleotides is negligible in comparison to the number of sequences we have generated, and other researchers are demonstrating that the random error of pyrosequencing is significantly less than any other sequencing technology.

Table S2. Number of homopolymeric tracts, and number of sequences containing them for each of the four marine virome libraries.

Library	*Number of tracts*	*Sequences with >1 tract*
GOM	1,659	1,650
BBC	4,053	3,832
Arctic	1,499	1,498
SAR	8,590	8,563
Total	15,801	15,543

Distribution of the marine viral sequences on the Phage Proteomic Tree

A new version of the Phage Proteomic Tree containing 510 phage genomes was constructed as described previously. All sequences were analyzed by tblastx against a database containing all of the completely sequenced phage genomes. Hits with an E-value $< 10^{-6}$ against this database (approximately equivalent to an E-value of 0.001 against the SEED nr database) were considered significant. For each sequence with a significant hit to the phage genome database, the top tblastx hit was recorded. To determine which phage phylogenetic groups were seen in each of the marine samples, each genome with at least one top significant hit in a particular sample was marked with a solid line in the corresponding column next to its position on the Phage Proteomic Tree. A version with the relative abundance of the phages was also constructed (Figure S2). In that case, the length of the bars is proportional to the relative abundance of the phage species in the community.

Analyses of the chp1-like Microphage

Please note: The SAR chp1-like Microphage is a consensus sequence constructed from the SAR metagenome. The input sequences actually represent a group of closely related viruses.

>SAR chp1-like Microphage

CCCAGCGTGCTGGGTTTAATCTCATGTCGTAGTGTAGTAAAGGTATCGTACCTTCCAAGCCACCGTTAGGAATAACATT
CTGTTCCATCATACCAAGGTATTCTGGACGTTGTAAACGTGCGTCTGGTGAGGTCACTCCGAAATGTGATTGTAATATTT
CGGTGTATCTTGTACCGCCTCGCGCGTCTTTCTCATAAAGACGTTGAATTTGAAACGCTTCGCGGAGTTCGTTAATTGTT
GCAGCTGCTGCATCTGTAAGATCTGCTGTAAAGTTGAAATCTTGCGCTGTTGCAAGGTTAGCTGCGCCTCCACCTGGGA
AAGTATTGGCGTAATAAAAAATCTTGGTTGTTAGATGCATCGTTATACATTGCGATGTATTTTCCATCGCCGGTAATTGA
TGACCCGCCAACTGCTGAGTAGGTAATTGGTGCTTCCGTTCCCAGTGGTAGTGTTACTGCATCGCCCTTTTGAGGGAAT
GGTAAACTGCTGGTAAAATAATCGTGGCGTTTACCACGCTTTTGTAGTGTGTAAGTTGTGTAAGTGTCCGGGCCATCGC
CTTTGTCTACTGTTAAGCTATCTTGTAGGTTTTTCGTCACGAAACCACTCATTCCATATAAGGTTATAAGCTCGTCCGTG
TAAGTTATTAAAATCTATACCAGCAATTTGCGTGGGAAGTCCCATGTAATCAAAAAGGCTATTTTCTGCCACCGTAGCT
CCGGTAATTTGAGGTACTAGAAAGTCTGTGCTATCGCCTGGATCGTCTTGTGCCCCGTTAAACTTTTCCCAATTGTCCCA
GATCAATCTATTGGGGACAAAGAAAAAGGAATGTCTCAACATACATATTATCCATAATTGGATATATCGGCGTTGCTAA
TCGACCAAAACCCTGTTGCGTTCATTTGAAAAGTATCGCCTGGTAGAACTTCGTCTACATAGATAGGGACCAGGTACCC
TGAATCGAATGTTGTCTTAAGGCCGTGCACACGGTTAAAGGTACTACGTTGAATATCCGCTTGTGGTACTCTGCTAAAT
TCGTGTGTAAGTGTAGTGGGTAGGCTACCCATTGGTCCGCCGAGCATATTAGTCTCCTAATGTTTCCATCTCKATAATTT
TCTTTGGTTTTTCCTGACCGGTAATTATTCCGGTCGTTTCGTCAAAGCTACCCAATCTGTGAAGCGAAAAATCGCTAGGG
TGCTTTGCGAATGCGTGATCCTTATTGTTGATCACTATGTCTTGAACCGCTCTTACTGCGGTTCCATCTTTAATCTCTAGA
AAGGGTTGTGAGTACATCTCAGCCTTTCTGTCATATACTGCGTAATAAACTTTCTTCATYTTTTCCTCCCGTGGAATATT
GTTACGGGAGAATCTTACGCATAATATACAATAGACGTCAATAGTTTATGTAACTGTTTGTTTGGACTCTTGTTACCCTG
TAAATGATTCATTTTATGACATTTTACAGGTTTCGAACTAATCGTTCGAGTTTTTTTATTTTTATTTCTTCTGACACCCAG
AGGTCATCCATCGCCTTTATTATATTCAATTATAGTCTCTGGCGCCTGTTCTTTTCGCTTCTCTTTCAGCTGCTCAAAGTA
TTCGGGATCGTGTTTCTGTAGTTCCTTATCGTAATACCTAGGAACTTTCATTTTTATACCATCGTGAACGATGTAATCGT
GCAAATGTGCATCTGTCCATCCGTATTTCCAATACCATTGATTTCCGATCCCGTTTTGAGGTTTTTKGTTTATTTCCACGC
GACATTGTCGCGTATTGGTTATCGAGATCGTATTCGATCTGACCTGTTTCGGGGTTTATATATTGCTCAGGGGGGCCCTC
CCCTTTCGCTCTTTTCATTACATACCGTGCCACATAATGGGCACTTTCGTATGTACACGCCCCAATTCTGTGGTAGCCGT
GGGGCCACAGTTCTTCTAATTCGGGTGATATATATAATTCGTTACCTAGTTTTTTTTCCCATAATTGTTTGTCTGGAAAAT
CATACCCGAATATTATTGCATGATAGTGGGGGCGTTTGTTTTCATCACCATATTCTCCGCAGTGAAAGAACTTAATGTCT
TTTCCTTTTTTTTTGCGGAGCCGTTTCAAAAATCTCTGAAACTCGGTGATGTCCAGAGACCAAGGGCGAGGGCGCTGTTC
AAGGGTCTCTGGGTTTATTGTTAAGGTTATGAAGCAATTGTGTTCGTGCATCTGGGCTTCATGCATACATCTGATAGCCC
ATTCACGACTGTGTTGCAGTCGCAACCCCAGCATTGACCACATGGAAGAATTAAAAGCCCTTTGCATATGCAAAGGGCT
TTAATCTTCCCTGTGGTCAGTGCTGGGGTTGCAGACTGCAACACAGTAGAGAATGGGCTATCAGATGTATGCATGAAGC
CCAGATGCACGAACACAATTGCTTCATAACCTTAACAATAAACCCAGAGACCCTTGAACAGCGCCCTCGCCCTTGGTCT
CTGGACATCACCGAGTTTCAGAGATTTTTTGAAACGGCTCCGCAAAAAAAAACAGAAAAGGACATTAAGTTCTTTCATTG
CGGAGAATATGGTGATGAAAACAAACGCCCCCACTATCATGCAATAATATTCGGGTATGATTTCCAGACAAACAATTA
TGGGAAAAAAAAACTAGGTAACGAATTATATATATCCCCCGAATTAGAAGAACTGTGGCCCCATGGCTACCACAGAAT
TGGGGCGTGTACATACGAAAGTGCCCATTATGTGGCACGATATGTTATGAAAAGAGCGAAAGGGGAGGGGCCCCCCTG
AGCAATATATAAACCCCGAAACAGGTCAGATCGAATACGATCTCGATAACCAATACGCGACAATGTCGCGTGGAAATA
AACAAAAAACCTCAAAACGGGATCGGAAATCAATGGTATTGGAAATACGGATGGACAGATGCACATTTGCACGATTAC
ATCGTTCACGATGGTATAAAAATGAAAGTTCCTAGGTATTACGATAAGGAACTGGAAAAATACGATCCTGAATACTTTC
AGGAATTGAAAGCGAAGCGGAAAGAACAGTCACCAGAGACTATAATAGAATATAATAAGGCGATGGATGACCTCTGG
GTGTCAGAAGAAATAAAAATAAAAAAACTCGAACGATTAGTTCGAAACTTGTAAAATGTCATAAAATGAATCATTTAC
AGGGTAACAAGAGTCCAAACAAACAGTTACATAAACTATTGACGTCTATTGTATATTATGCGTAAGATTCTCCCGTAAC
AATATTCCACGGGAGGAAAAGATGAAGAAAGTATATTACGCAGTGTATGACAGAAAAGCAGAGATGTATTCACAGCCT
TTTCTAGAGATAAAAGACGGTACAGCAATAAGGGCTGTTCAGGACATAGTAATCAACAGTAAAGACCATGCGTTCGCA
AAACATCCCAGAGATTTCACATTATTCAGACTGGGTGAATTTGACGAAACGACAGGCGTAATAACCGGACAGGATAAA
CCGAAACAGATCATAGAGATTGAAACACTTGGAGAGTTAAAAAATGCTAGGCGGACCAATGGGCACCCTGCCCACCAC
ATTATCACACGAATTCTCACGCGTACCTCAAGCAGATATTCAACGTAGTACCTTTAACCGTGTACACGGGCTTAAAACA
ACATTCGATAGTGGATACTTGGTTCCGATATTCGTCGACGAAGTTCTCCCCGGCGATACGTTTCAATGTAGCGCGACGG
GCCTTTGGTCGCCTTTCAACTCCTCTCTACCCAGTAATGGATAACATGTATGTAGAAACATTCTTTTTCTACGTCCCAAA
TCGTATTATCTGGGACAACTGGGAGAAACTCAACGGTGCACAGGATGATCCGAACGACAGTACAGATTTTCTGGTTCCC
CAAATACAATCGGCAACAATAGCTCAGGATACTCTTTTCGATTATATGGGACTTCCCACCAAGACAGCAGGTTTGAACT
TTAACAACCTGCACGGTAGAGCATACAACCTCATCTGGAACGAATGGTTCCGAGATGAAAATTTACAGGATTCCCTAGT
AGTAGATAAGGACGATGGCCCTGACACTTTAACAGATTATACACTACAAAAAACGTGGTAAAAGACACGATTATTTTA
CCTCTGCCCTACCATGGCCTCAGAAAGGCGATGCAGTAAACCTACCACTCGGAACATCTGCTCCAGTAGCAACGGATTC
CGCAGATGGTGAAAACATAGCAGTATATTCAACAGGATTAGGCGGCTATACCAATATGGCGGCGAATGGAACCTTTGT
GGAAAACCCGTTCGGCGGTGGAACCGAAGACCGCTCACTATATGCCGACCTAACAGATGCAACAGCAGCAACAATCAA
CGAATACGCGAAGCGTTTCAAATCCAGAGACTTCTGGAGCGTGACGCTAGGGGCGGCACAAGATATACCGAGATTTTA
CAATCCCATTTTGGAGTAACCTCACCAGACGCCGCTTACAGCGTCCGAGTATCTCGGCGGCTCAAAAACAGAAATAAA
CATGCAGCCAATTCCACAGACTGGTTCAACAGACAGTACATCTCCTCAAGGTAACCTAGCAGCAATAGGTACAGCATC
ATCCAGAGGCGGATTTTAATAAGTCTTTTGTAGAACATGGTGTAATTATCGGAATGGCATGCGTATTTGCAGACTTAAC
TTATCAACAAGGGTATGAACCGTATGTGGTCACGTCGTGACCGCTGGGACTTTTATTGGCCAGCTCTCGCCCATTTAGG
CGAACAAGCGGTTCTAAACCAAGAAATCTATTATCAAAACACTTCAGCGGATTCCCAGACCTTTGGCTATCAGGAACGC
TGGGCAGAATATAGATATAAACCAAGCCAGATCACTGGCAAAATGCGTTCGAACGCAACAGGCACCTTTAGACGTATG
GCACTTGGCACAGGATTTCTCCTCGCCTGCCGGCACTCAACTCTTCATTCATCGAAGAAAACCCACCCATCGATCGGGT
TATCGCAGTAACCGACGAACCACAATTCATCTGGGACTGGTACTTCGATCTTAAATGTACAAGACCAATGCCTGTTTAT
TCAGTACCAGGCTTAATCGATCACTTCTAGGTGCAATATGAATGGATTCAAGTGGACCATTATTCTTGGCGTTCTTCGCA
AGTATGC

>chlamyd4 (Chlamydia phage 4; AY769964.1)

ATGGTTAGGAATCGGCGTTTGCCTTCAGTTATGAGTCATTCTTTCGCGCAAGTCCCATCAGCGCGAATTCAGAGAAGTT
CTTTTGATAGATCTTGTGGTTTAAAAACTACATTCGACGCCGGTTACCTAATCCCTATCTTTTGTGATGAAGTTCTCCCT
GGAGATACTTTCTCCTTGAAAGAGGCGTTTTTAGCACGTATGGCAACGCCTATCTTTCCTCTTATGGATAATTTGCGTTT
AGATACGCAGTATTTCTTTGTTCCTCTTCGACTATTATGGTCGAATTTTCAAAAGTTCTGTGGAGAACAAGATGATCCTG
GAGATTCTACAGATTTTCTTACCCCAATTTTGACCGCTCCTCAGAATGGTGGTTTTGCTGAAGGATCGATCCATGATTAT
CTTGGTCTACCTACTAAAGTTGCAGGAGTTCAATGTGTTGCGTTTTGGCACAGAGCTTACAATTTGATTTGGAACCAGTA
CTATCGTGATGAAAATATTCAGGATTCAGTTGAAGTGCAAATGGGAGATACCACTGCAGATGAAGTGAACAATTATAA
GCTTCTTAAGCGCGGGAAGCGTTATGATTATTTCACTTCATGTCTCCCTTGGCCACAAAAAGGTCCTGCAGTGACAATC
GGAGTTGGAGGTATTGTTCCTGTTCAAGGTTTAGGAATTCAATGGGGCGGTTCTACAGGTCCAAATCCTATAACTGCTT
CTGATTGGAGAGATTCCGTTAATCCTACATATGTAAATTCTGCAACGCAGACGCCTACAGGAACGAATAAGATTTTGAG
TTATGGTCAGGCGTATTATATTAAGAAGCCTGGAGAACCAGCTACAGATCCTGCACCTAGGGCTTATGTAGATTTAGGT
TCGACTTCTCCTGTGACGATTAATTCTCTTCGTGAAGCTTTCCAATTGCAAAAGCTTTATGAGAGAGATGCCCGTGGTGG
AACAAGGTACATTGAGATTATTCGTTCCCATTTCAATGTGCAGTCTCCAGATGCAAGGTTGCAACGTGCAGAGTATCTT
GGAGGTTCTTCAACTCCTGTGAATATTTCTCCGATTCCACAGACTTCCTCAACAGACTCCACATCTCCTCAAGGAAATCT
TGCTGCTTATGGTACAGCGATTGGATCGAAGCGAGTCTTCACAAAGTCCTTCACAGAACATGGTGTAATCCTTGGATTA
GCCTCTGTACGCGCCGATCTCAACTATCAGCAAGGTTTGGATAGGATGTGGTCACGAAGAACGCGCTGGGACTTTTACT
GGCCTGCTCTTAGCCATTTAGGTGAGCAAGCTGTGCTCAATAAAGAGATCTATTGCCAAGGTCCTGCAGTTAAGGATGC
TCAGAATGGCAATGTTGTTGTGGATGAGCAAGTCTTTGGATATCAGGAGAGATTTGCGGAGTATCGCTATAAGACTTCG
AAAATTACTGGCAAGTTCCGATCAAATGCTACAAGTTCTTTAGATTCATGGCATTTAGCTCAGGAATTTGAGAATCTTC
CAACACTTTCTCCGGAGTTTATCGAAGAAAATCCTCCTATGGATCGTGTTCTTGCTGTAAATACTGAGCCAGATTTTCTT
TTAGATGGCTGGTTTTCATTGCGTTGTGCAAGACCAATGCCTGTCTACTCTGTTCCAGGCCTCATTGATCATTTCTAATTT
CTACTCAGTTTTCCGATTTGATAAAGCAAACTCACGTTCGTAGATAAGTGAGTACGGTGAAGACCAAAACGGAAAGCT
GAGGCGTAAAAATGTGGAGAATTTATGAATCCCGAACAACTTACGAACACTCTCGGTTCAGCAGTTTCTGGAGTTGCGC
AAGGATTATCCTTTCTCCCTGGAATAGCTTCCGGAGTTTTAGGATATCTTGGTGCACAAAAGCAAAATGCCACTGCGAA
GCAAATTGCTAGAGAGCAAATGGCTTTTCAGGAGCGCATGTCTAACACGGCATACCAACGTGCCATGGAAGACATGAA
GAAAGCTGGCCTTAACCCTATGTTAGCTTTTTCTAAAGGCGGTGCTTCTTCTCCTGCAGGAGCGTCATGGTCTCCGAATA
ATCCTGTAGAAAATGCGATGAATTCTGGCCTTGCCGTGCAAAGACTTACTTACGAACGTAAGAAAATGCAGGCAGAGC
TTCAGAATCTTCGTGAGCAGAACCGTTTGATTAGAAATCAAGCAATACGTGAAGGCTATCTCGCAGAACGAGATAAAT
ATATGCGTGTTGCTGGAGTTCCTGTGGCCACTGAGATGTTAGATAAGACTTCTGGTCTTATCTCATCTTCAGCTAAGGCA
TTTAAGAATCTTTTTTCAAGAAAAGGAAGGTAGATGTTTAAGTCGGCATATTCCGAAAAAAAATCTGTAAAGATGAAGT
TCACACAGAAATCTTTGACGCAGCAACACAACAAAGATGAGTGTGATATTAACAACATCGTCGCAAAACTCAACGCTA
CAGGCGTTTTAGAGCACGTAGAGCGACGATCTCCACGTTATATGGACTGTATGGACCCTATGGAGTATTCCGAGGCTCT
AAACGTCGTTATTGAGGCTCAGGAGCAATTTGACTCTTTACCAGCCAAAATTCGTGAACGTTTTGGAAATGATCCAGAA
GCGATGCTCGATTTCTTGAGCCGTGAAGAAAATTATGAAGAAGCAAAGGCGTTAGGTTTTGTTTATGAAGATGGAACTT
CTGGAGCACCTCAAACATTTTTTGAAGCTGATCCTAAAGATGATCAAAATGTGGCAAACCAAGAACCTGGATTAGCCC
AAAAATGAGCAAATTTTGTGCAAAAAAGTGTGCAAAAAAAATGTGCAAAAAAATGGGCCAAAAATTGCCCCCAAAATCG
GAGCATTTTACGAGAGAAAAACACCAGCGTGTAACAGTCTTACTTGATCTGTTACACGCCTGGTGGTCGGAATTGTAAG
GAAATTTTTTAAAACTAAGCCCTATTTAGGGCCCAAAATTTAAGCTTAAAATGAGGTTAAAAAATGGCACGAAGATAC
AGACTTTCGCGACGCAGAAGTCGACGACTTTTTTCAAGAACTGCATTAAGAATGCATCGAAGAAATAGACTTCGAAGA
ATTATGCGTGGCGGCATTAGGTTTTAGTTTTGGATGTTAAGGAAATCTTTAAGGTTATGCTAAATTAGCTGCTATGTATA
ATTTGGCTCGTGACGAATGTATGTCATATTCGCACCGTTTACAATTACACAGCAGTTGAAGGCTTAGACGTTGATTTTTA
ATGTCTTAGCCTTCATTTTTGGTTTAGTGTGATTGCAAATGAGGTGCTCATGACGTGCATTTCTCCTTTTGTATGTTTTAT
AGATCCTTGTAACCAGCTCTGGTTTCCCAAAGGTGAGAAGTCTTCTAAACCTTGGGATAAAGTCCGTGAATTAAATGCT
TTTGAGCAAACGCAACCTGAAGAGTATCGAAAACGTTGGATTTTGATGCCTTGCCGTAGGTGCAAGTTTTGTAGAGTGC
AGAATGCAAAGATTTGGTCGTATCGTTGCATGCACGAAGCGTCTTTATATTCTCAGAATTGCTTTTTAACTTTGACTTAT
GAGGATCAGCATCTTCCAGAGAATGGTTCTCTGGTAAGAAATCATCCGACTTTGTTTCTTAGGCGATTGAGAGAGCACA
TTTCTCCTCATAAGATTCGTTATTTTGGATGTGGTGAATATGGATCGAAATTACAAAGGCCTCATTATCATCTTCTTATTT
ATAATTACGATTTTCCTGATAAAAAGCTCTTGAGTAAAAAGCGTGGCAATCCTCTCTTTGTTTCTGAGAAGTTAATGCA
GCTTTGGCCGTATGGATTCTCTACAGTGGGATCTGTAACGCGGCAAAGTGCAGGTTATGTAGCGCGCTATTCTTTGAAG
AAAGTGAGTAGAGATATTTCTCAAGATCATTATGGTCAAAGACTTCCGGAGTTTCTTATGTGTTCTCTTAAACCAGGAA
TAGGAGCGGATTGGTATGAGAAATATAAACGCGATGTCTATCCTCAGGATTATCTTGTTGTGCAAGATAAAGGGAAGT
CTTTTACGACGCGTCCTCCACGTTACTATGATAGCTACATTCTCGGTTTGATCCGGAAGAGATGGACGAGGTCAAACAA
AAACGTGTAGAGAAAGTCATGGCTTTGCCTGAGCTATCTCAGGATAAGGCTGAGGTGAAGCAATATATTTTCAATGACC
GTACGAAGAGACTCTTTAGAGACTATGAGGAGGAGAGTTACTAAACTTTTTTAAAAAATAGGAGCTTTTTTCAATGAAA
GTTTTTACAGTGTTTGATATTAAGACGGAAATTTATCAGCAGCCTTTTTTTATGCAGGCTACGGGAGCGGCAATCAGAG
CGTTTTCCGATATGGTAAATGAGGATCCTACAAAGAATCAATTTGCCGCGCATCCTGAAGATTACATTCTCTATGAGAT
TGGATCTTACGATGACTCTACTGGAACTTTCATTCCCTTAGATGTGCCTAAAGCCTTAGGAACAGGCTTGGATTTTAAGC
ACAAACAGTAGGGAAGAT

Global sample diversity and cross-contig simulation

For these analyses, 2500 random sequences were taken from each of the 4 metagenomes, totaling 10,000 sequences. This is the **mixed sample**. The sequences were

assembled with TIGR_Assembler using a minimum of 98% identity over at least 20 bp and no sequence alignment error in 32 bp ("-g 1" argument).

A normal **contig spectrum** was determined by counting the number of *q*-contigs, where *q* is the number of fragments in any particular contig (Figure S3).

For a particular set of sequences, the average fragment length was 102 bp. All of the contigs of 5 or more sequences (i.e., q>4) only contained sequences from one library and the contig spectrum from the mixed sample was:

Mixed contig spectrum: [9474 130 26 13 7 5 2 0 2 1 0 0 0 1 1 0 0 0 0 0 0 0 0 0 0]

q	#	Samples that each contig came from
15	1	1 GOM – i.e., all 15 sequences that assembled were from GOM
14	1	1 GOM
10	1	1 GOM
9	2	1 BBC, 1 SAR
7	2	1 GOM, 1 BBC
6	5	2 GOM, 1 BBC, 2 SAR
5	7	4 GOM, 3 SAR
4	13	5 GOM, 3 BBC, 4 SAR, 1 cross
3	26	11 GOM, 1 Arctic, 1 cross
2	130	36 GOM, 10 BBC, 24 Arctic, 41 SAR, 19 cross
1	9474	singletons: 2297 GOM, 2446 BBC, 449 Arctic, 2324 SAR

q = number of fragments in each contig (size of the contig)

# = number of contigs

To determine a **cross contig spectrum**, only sequences that assembled with sequences from other regions were kept. The number of *q*-cross-contigs was then counted as the number of remaining contigs of *q* sequences. The total number of singletons (1-contigs) from each region that assembled with any fragments from other regions was the number of 1-cross-contigs. The method to determine this cross-contig spectrum is represented in Figure S4. In the example above, the cross-contig spectrum was: [42 19 1 1 0]

Dissolved contig spectra were calculated for each separate metagenome by determining how many of the contigs came from only one metagenome. The dissolved contig spectra were not used in the manuscript except as a check on the methodology (i.e., they should be similar to contig spectra obtained when assembling individual metagenomes).

After repeating the process 10 times to get a better coverage of the metagenomes, the resulting contig spectra were averaged yielding the following:
Average mixed contig spectrum: [8870.1 227.5 49.9 23.4 11.8 7.4 4.3 3.2 2.8 1.3 1.7 1.2 0.7 0.5 0.9 0.4 1 0.4 0.6 0.4 0.3 0.2 0.1 0.2 0.1 0.1 0.2 0.2 0.1 0.2 0.2 0.3 0.2 0 0.3 0.1 0 0.1 0.3 0.2 0 0 0.1 0 0 0 0 0.2 0.1 0 0 0 0 0 0 0 0 0 0]
Average cross-contig spectrum: [48.9 23.5 1.4 0.2]

To estimate community structure and diversity, the averaged mixed contig spectrum was analyzed using **PHACCS** (http://phage.sdsu.edu/research/tools/phaccs/) using the following parameters for the example above: 102 bp for the average fragment size, an average genome length of 50 kb, and looking for up to 100,000 genotypes. The results are presented in Table S3.

Table S3. Example of PHACCS output using the average mixed contig spectrum mentioned above. The best fit (lowest error) in this example was for a logarithmic distribution of the genotypes.

	Error	Richness	Evenness	% most abundant	Shannon (nats)
**Power law**	4560.1	100,000+ *			
**Exponential**	26,208	10,001	NaN	8.3849	NaN
**Logarithmic**	2324.8	57,572	0.89481	9.3394	9.8078
**Lognormal**	3906.3	100,000+ *			
**Niche premption**	26,208	10,001	NaN	8.3849	NaN
**Broken stick**	20,095	53	0.89884	8.5979	3.5687

* 100,000+ means that the best parameters for the tested distribution were not found by PHACCS using the specified input parameters.

The **Monte Carlo simulation** was used to determine whether differences between observed viruses within a community are due to changes in their relative rank (i.e., the abundance they make in the community) or because they are fundamentally different viruses (illustrated in Figure S5).

The average cross-contig spectra were then compared with simulated average cross contig spectra from simulated mixtures of the four communities (Figure S6).
